# Robot-Assisted Electrode Array Insertion Becomes Available in Pediatric Cochlear Implant Recipients: First Report and an Intra-Individual Study

**DOI:** 10.3389/fsurg.2021.695728

**Published:** 2021-07-07

**Authors:** Huan Jia, Jinxi Pan, Wenxi Gu, Haoyue Tan, Ying Chen, Zhihua Zhang, Mengda Jiang, Yun Li, Olivier Sterkers, Hao Wu

**Affiliations:** ^1^Department of Otorhinolaryngology Head and Neck Surgery, Shanghai Ninth People's Hospital, Shanghai Jiaotong University School of Medicine, Shanghai, China; ^2^Ear Institute, Shanghai Jiaotong University School of Medicine, Shanghai, China; ^3^Shanghai Key Laboratory of Translational Medicine on Ear and Nose Diseases, Shanghai, China; ^4^Department of Radiology, Shanghai Ninth People's Hospital, Shanghai Jiao Tong University School of Medicine, Shanghai, China; ^5^APHP, Groupe hospitalo-Universitaire Pitié Salpêtrière, Otorhinolaryngology Department, Unit of Otology, Auditory Implants and Skull Base Surgery, Paris, France

**Keywords:** cochlear implant, robot-assisted, robotic, hearing loss, scalar position

## Abstract

**Background:** As an advanced surgical technique to reduce trauma to the inner ear, robot-assisted electrode array (EA) insertion has been applied in adult cochlear implantation (CI) and was approved as a safe surgical procedure that could result in better outcomes. As the mastoid and temporal bones are generally smaller in children, which would increase the difficulty for robot-assisted manipulation, the clinical application of these systems for CI in children has not been reported. Given that the pediatric candidate is the main population, we aim to investigate the safety and reliability of robot-assisted techniques in pediatric cochlear implantation.

**Methods:** Retrospective cohort study at a referral center in Shanghai including all patients of simultaneous bilateral CI with robotic assistance on one side (RobOtol® system, Collin ORL, Bagneux, France), and manual insertion on the other (same brand of EA and CI in both side), from December 2019 to June 2020. The surgical outcomes, radiological measurements (EA positioning, EA insertion depth, mastoidectomy size), and audiological outcomes (Behavior pure-tone audiometry) were evaluated.

**Results:** Five infants (17.8 ± 13.5 months, ranging from 10 to 42 months) and an adult (39 years old) were enrolled in this study. Both perimodiolar and lateral wall EAs were included. The robot-assisted EA insertion was successfully performed in all cases, although the surgical zone in infants was about half the size in adults, and no difference was observed in mastoidectomy size between robot-assisted and manual insertion sides (*p* = 0.219). The insertion depths of EA with two techniques were similar (*P* = 0.583). The robot-assisted technique showed no scalar deviation, but scalar deviation occurred for one manually inserted pre-curved EA (16%). Early auditory performance was similar to both techniques.

**Conclusion:** Robot-assisted technique for EA insertion is approved to be used safely and reliably in children, which is possible and potential for better scalar positioning and might improve long-term auditory outcome. Standard mastoidectomy size was enough for robot-assisted technique. This first study marks the arrival of the era of robotic CI for all ages.

## Introduction

Minimizing intracochlear trauma is an essential consideration in cochlear implantation (CI), particularly the preservation of residual hearing, by applying soft surgery and derivative techniques (1–5). As a key procedure in CI, the electrode array (EA) should be placed in the scala tympani and avoid damaging the intracochlear structures. Positioning the EA in the scala tympani leads to better postoperative speech recognition compared to the outcomes with scala vestibuli insertion. Because scalar deviation or translocation can increase the distance between the electrode and the ganglion cells, decreasing the electrical stimulation efficiency, and damage the basilar membrane, inducing residual hearing loss (6, 7). Despite the improvements in EA design and surgical approach, the incidence of EA deviation or translocation is very common, especially with perimodiolar arrays (8). Besides the experience of the surgeon, the natural limitations of the human hand, such as tremor, drift, or jerk, appear to be the main factors leading to intracochlear damage (9, 10).

To overcome the bottleneck in manual micro-manipulation, several otological robots have been designed and applied in CI, the main ones being RobOtol® (Collin ORL, Bagneux, France) by Sorbonne University/AP-HP (11), HEARO® (CAScination AG, Bern, Switzerland) by Bern University (12), and micro-stereotactic frames by Vanderbilt University (13). Among them, RobOtol® focuses on minimally invasive insertion of the EA, and previous studies in temporal bones and animal models have shown that semi-automated robot-assisted insertion was more accurate and less traumatic than manual insertion with a higher number of electrodes correctly positioned in the scala tympani (14, 15).

As the first device to obtain European certification for clinical use (CE mark), the RobOtol® system has been used in France and China since 2019 for robotic-assisted CI in profoundly deaf adults (16–18). Both teams reported that robot-assisted EA insertion was a safe surgical procedure. As the mastoid and temporal bones are generally smaller in children, which would increase the difficulty for robot-assisted EA insertion, the clinical application of these systems for CI in children has not been reported.

After successful use of RobOtol® in adult CI recipients and with safety verification in pediatric temporal bone models (PHACON Temporal Bone Patient “Klein,” GA, USA), our center has performed robotic-assisted EA insertion in children since December 2019. Here we present the first series of pediatric CI with robotic assistance reporting the efficacy of robot-assisted insertion in children and clarifying the surgical safety issues of RobOtol® in pediatric patients.

## Materials and Methods

### Study Design and Participants

We conducted a retrospective cohort study of simultaneous bilateral CI recipients from December 2019 to June 2020 at our department (SH9H-2020-T166-1). Inclusion criteria were: (1) same brand of CI and EA on both sides; (2) unilateral robotic and contralateral manual EA insertion. Five infants and an adult were enrolled in this study.

Patient demographics, information on the cochlear implant, medical records, radiological data, and audiological outcomes were collected. All parents of infants and adult patient gave their written informed consent to permit the use of their medical data.

### Cochlear Implants

Three types of cochlear implants were used: (i) Cochlear CI512 with a perimodiolar array of 22 electrodes, 19/15.6 mm length (total/active), 0.4 mm diameter in the distal part, and 0.8 mm in the proximal portion (Cochlear AG, Lane Cove, Australia); (ii) Concerto FLEXsoft with a lateral wall array of 19 electrodes, 31.5/26.4 mm length (total/active), 0.5 × 0.4 mm diameter in the distal part and 1.3 mm in the proximal portion (MED-EL, Vienna, Austria); (iii) CS-10A TM with a lateral wall array of 24 electrodes, 22/20 mm length (total/active), 0.4 mm diameter in the distal part and 0.8 mm in the proximal portion (Nurotron, Hangzhou, China) (19).

### Surgical Techniques

In all cases, the CI followed the soft surgery protocol (1) and was performed by two senior otologists (manual insertions by H.W.; robot-assisted insertions by H.J.). The same standard surgical approach was used for both insertion techniques: a retro-auricular approach, mastoidectomy, posterior tympanotomy, and exposure of the round window. After opening the round window, either manual or robot-assisted insertion was applied by one right-handed senior otologist via a round window approach. The manual insertion was slowly and carefully performed according to the minimally invasive protocol (20–23).

**Figure 1:**
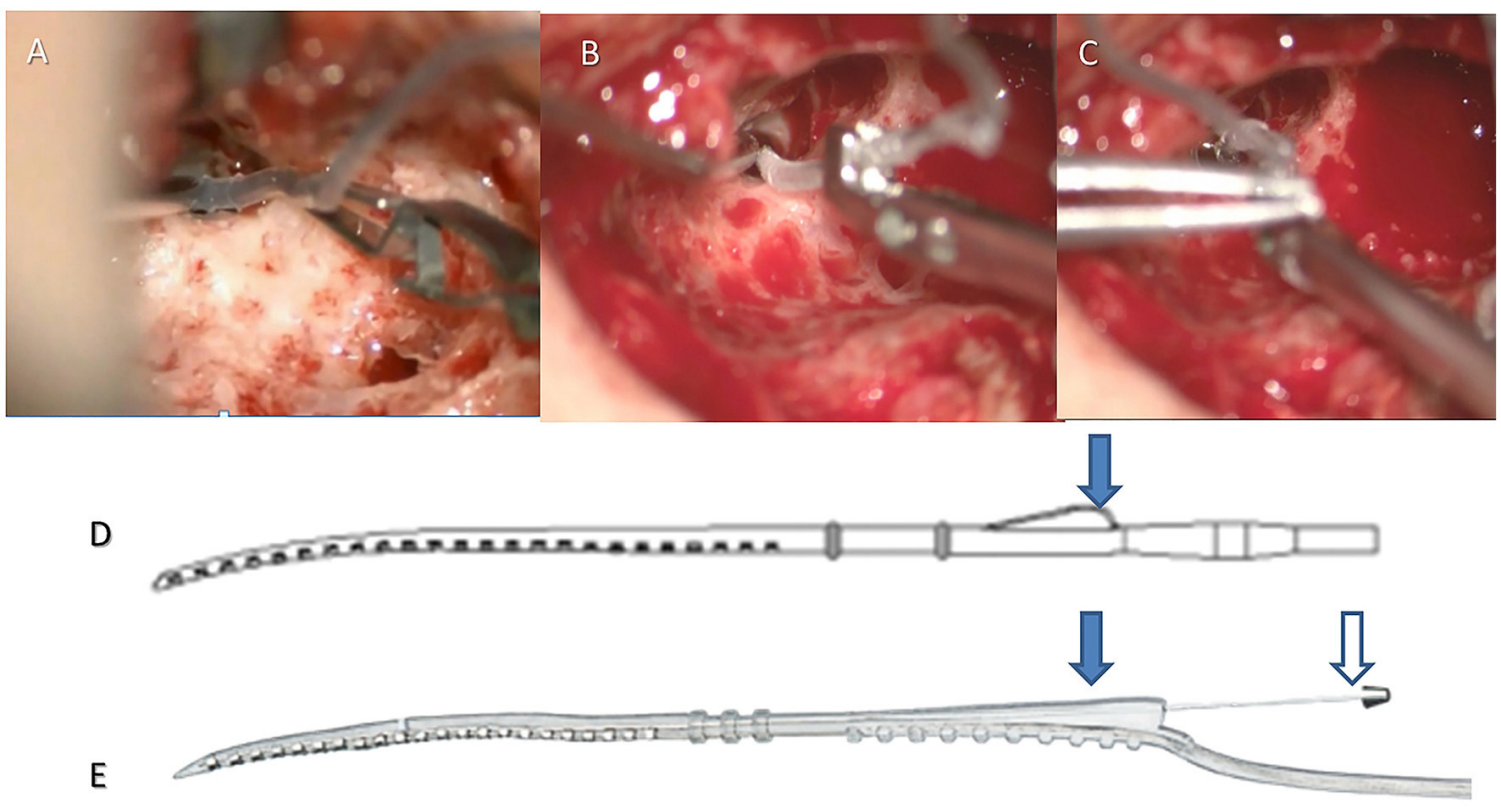
Robot-assisted insertion with a lateral wall or perimodiolar electrode array. For the lateral wall EA (e.g., CS-10A TM), the crest of the EA (arrow in **D**) was clamped by the robotic tool **(A)**, the EA was robotically advanced until the round window marker reached the round window, then the EA was released. For the perimodiolar EA, the crest was clamped by the robotic tool (arrow on the left in **E**), the EA was robotically advanced until the first marker reached the round window **(B)**, then the stylet was held manually (arrow on the right in **E**). The EA was then robotically advanced until the round window marker reached the round window **(C)**, then the stylet was manually retracted, and finally, the EA was robotically released (Robot-assisted AOS technique).

Robot-assisted EA insertion was performed using the RobOtol® system as a platform, and a specific custom-made micro-forceps (Nurotron, Hangzhou, China) as an actuator (Figure 1). The speed of the robotic arm could be switched between three gears (high speed: 10 mm/s max; medium speed: 2 mm/s max; low speed: 1 mm/s max). Before the robot-assisted procedure, RobOtol® was draped with a sterile cover, moved into the optimal surgical position, and then the sterilized micro-forceps was mounted on the arm (16–18). The surgeon controlled the robot-assisted arm with the SpaceMouse (3D-connection, Waltham, MA, USA) mounted on the rail of the operating table. The closing and opening of the micro-forceps were controlled by two buttons on the SpaceMouse. After adjusting the robotic arm to the optimum position and aiming it at the ideal insertion axis, the EA was introduced slowly into the cochlear through the round window (low-speed mode), advancing to the target position without interruption, and then released carefully (Figure 1). For the perimodiolar EA, the stylet was manually held and later extracted using the Advance Off-Stylet (AOS) technique (Figures 1B,C,E) (24). The same standard technique for closure of the surgical cavity was applied on both sides. The duration of the following procedures was recorded:

1) Robotic arm preparation time: moving the robotic system into place and adjusting its arm to the surgical field (additional time required compared to classic manual surgery);
2) EA preparation time for robotic assistance: mounting the EA on the robotic tool, opening the round window, and aiming the robotic arm along the insertion axis;
3) EA insertion (either manual or robotic).

### Radiological Analysis

**Figure 2:**
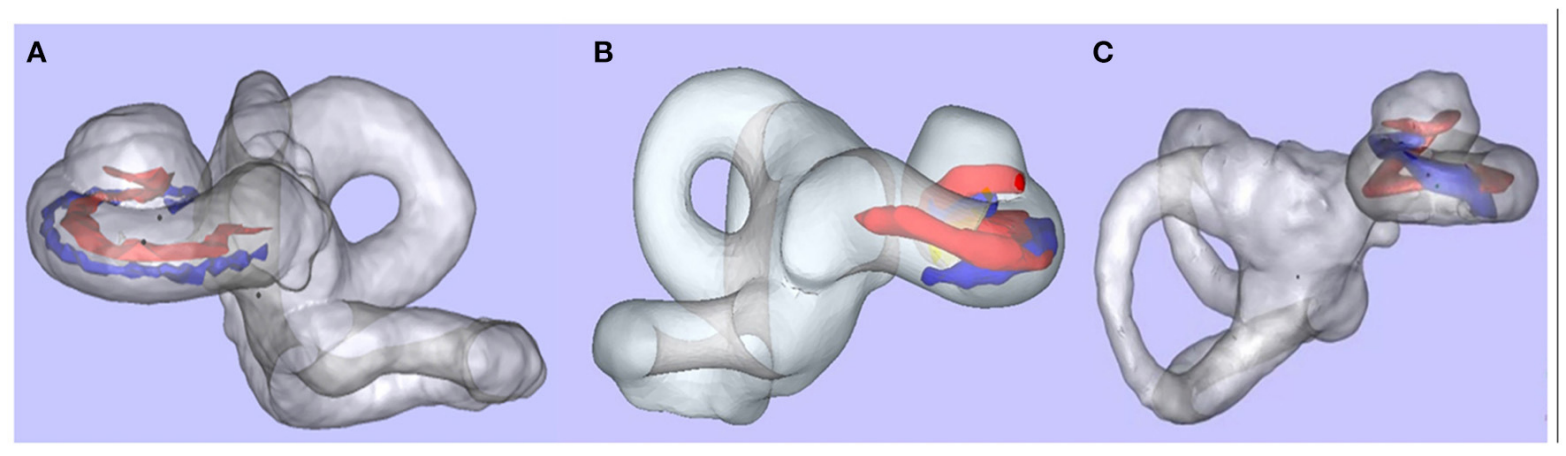
Scalar positioning of the electrode array. After 3D merged reconstruction, the cochlea (membrane labyrinth, gray), the basilar membrane (red), and the EA (blue) could be observed clearly. **(A)** Full scala tympani positioning of EA. **(B)** Scalar deviation at 180°. **(C)** Scalar translocation from 180° (data from other patient, not in this study).

All patients underwent preoperative high-resolution spiral computed tomography (CT), magnetic resonance imaging (MRI), and a post-implantation (spiral or cone-beam) CT. The cochlear length (distance A), width (distance B), and height (distance H) were measured by Otoplan (CAScination AG, Bern, Switzerland) on the preoperative CT image and completed by the audiologist (Y.C.) independently. The insertion depth and the number of extracochlear electrodes were measured, and the scalar positioning of the EA was assessed by 3D fusion reconstruction of pre-and post-implantation CT with itk-SNAP, CloudCompare, and Blender, as previously described (25). This evaluation was performed by an otologist (H.T.) and a neuroradiologist (M.J.), blinded to the treatment allocation. Full scala tympani positioning of EA was shown in Figure 2A. “Scalar deviation” (Figure 2B) was defined as the presence of at least one electrode located above the basilar membrane although the distal electrode returned into the scala tympani. “Scalar translocation” (Figure 2C) was defined as the presence of one or several electrodes located above the basilar membrane from the penetration site to the tip of the EA.

**Figure 3:**
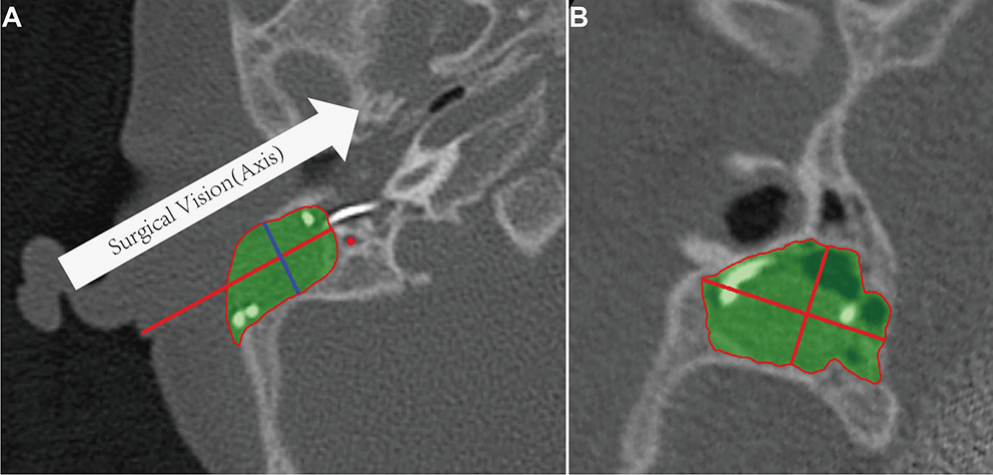
Mastoidectomy size and related anatomic parameters. **(A)** Definition of surgical vision (axis) on the axial plane. The green portion is the volume of the mastoidectomy, the red line is the distance from skin to posterior tympanotomy. **(B)** The area, transverse length, and longitudinal width of the maximum cross-section (blue line in **A**) in the direction of surgical vision.

The measurement of mastoidectomy size parameters was realized using Mimics 17.0 (The Materialise Group, Leuven, Belgium) by removing the postoperative 3D temporal bone volume from the preoperative volume (Boolean operation). The direction of the surgeon's sight, which is parallel to the posterior wall of the external auditory canal on the axial plane, was defined as the axis of surgery. The cross-section of the mastoidectomy was vertical to this axis. The maximum cross-section along this axis was defined as the surgical vision plane, and its transverse length, longitudinal width, and area were measured (Figure 3). The distance from skin to facial recess on this axis was also measured as the depth. Two researchers from the hospital cross-checked the measured data for quality control.

### Audiological Evaluations

The preoperative audiological evaluation included the click and tone-burst auditory brainstem response (ABR) in pediatric cases to estimate the corresponding audiometry thresholds in children (26), and additionally, pure-tone audiometry and speech discrimination score (SDS) with Mandarin speech test materials (MSTMs) in a soundproof room for the adult case. The mean threshold of audiometry was calculated at 0.5, 1, 2, and 4 kHz. The first mapping of CIs was performed 1 month after surgery, and subsequent mapping was performed regularly at our center. Postoperative auditory outcomes were collected at 6 months after first mapping. Behavioral audiometry for the infants and aided hearing thresholds for the adult at 0.5, 1, 2, and 4 kHz were recorded.

### Statistical Analysis

SPSS statistical software, version 20 (IBM Corp., Armonk, NY, USA) was used for statistical analysis. No imputation was made for missing data. Values are presented as means ± standard deviation (SD). Auditory outcomes and anatomic measurements for the two sides were analyzed using a paired-samples *t*-test or Wilcoxon rank-sum test. A *P*-value < 0.05 was considered statistically significant.

## Results

In this study, the five infants were 17.8 ± 13.5 months old (10–42 months), and the adult was 39 years old at the time of surgery. All patients experienced severe or profound hearing loss (HL), and no syndromic deafness was found. The infants all had congenital HL, and the adult presented progressive HL for 17 years with profound HL for 2 years and with no benefit from hearing aids. No inner ear malformation was observed. The distances A, B, and H of the bilateral cochlea in these cases were similar. Two cases were bilaterally and simultaneously implanted with the FLEXsoft lateral wall EA, two cases with the CS-10A TM lateral wall EA, and two cases with the CI512 perimodiolar EA.

Both insertion techniques were successful without intraoperative complications. Intraoperative electrophysiological measurements such as the electrodes impedance and neural response telemetry (NRT) thresholds were normal in all ears. When using the RobOtol® system, the extra preparation time to position the robotic arm was 208.2 ± 105.6 s, and the additional preparation time to position the EA was 241.7 ± 123.5 s. The duration of insertion under robotic assistance was 197.8 ± 64.5 s, which was significantly slower than that by manual insertion (72.8 ± 10.1 s, *n* = 6, *t* = 5.39181, *p* = 0.003) (Table 1). With robotic assistance, the FLEXsoft EA seemed to require a longer preparation time which took an average of 386 s compared to an average of 191 and 148 s for the CS-10A TM and CI512 EAs, respectively. The insertion times were shorter with the perimodiolar EA, taking an average of 127 s compared to an average of 269.5 and 197 s for FLEXsoft and CS-10A TM EAs, respectively.

**Table 1:** Cochlear implant type and surgical outcomes in the six cases in this study.

**Sex**	**Age at surgery**	**Device**			**Surgical technique**				**EA positioning**		
		**Model**	**EA type, total/active length (mm)**	**Side**	**Robotic arm preparation time (s)**	**EA preparation time (s)**	**EA insertion time (s)**		**Insertion depth (°)**		**Scalar position**
M	13 mo	Concerto FLEXsoft	Lateral wall, 31.5/26.4	**R**	**371**	**440**	**281**		**579**		**All in ST**
				M				87		583	All in ST
M	13 mo	Nurotron CS-10A TM	Lateral wall, 22/20	**R**	**145**	**241**	**188**		**406**		**All in ST**
				M				80		429	All in ST
F	10 mo	Concerto FLEXsoft	Lateral wall, 31.5/26.4	**R**	**306**	**332**	**258**		**588**		**All in ST**
				M				78		591	All in ST
F	42 mo	Cochlear CI512	Perimodiolar, 19/15.6	**R**	**105**	**125**	**132**		**387**		**All in ST**
				M				63		376	All in ST
M	12 mo	Cochlear CI512	Perimodiolar 19/15.6	**R**	**178**	**171**	**122**		**377**		**All in ST**
				M				65		349	In ST, except scalar deviation at 180–210°
*F*	39 yr	Nurotron CS-10A TM	Lateral wall, 22/20	***R***	***144***	***141***	***206***		***444***		***All in ST***
				*M*				*64*		*427*	*All in ST*

*Sex column: M, male; F, female. Age column: mo, month; yr, year. Side column: R, inserted with robot assistance; M, inserted manually. EA, electrode array. ST, scala tympani. Boldface indicates the robot-assisted side. Italics indicates the adult case*.

There were no postoperative complications such as local infection or facial palsy in these cases. Postoperative imaging showed full insertion of the EA in all cases. There was no difference in insertion depth between the robot-assisted and manual insertion sides (*t* = 0.58692, *p* = 0.583). The average insertion depth was 585.3 ± 5.3° for the Med-El lateral wall EA, 372.3 ± 16.3° for the Cochlear perimodiolar EA, and 426.5 ± 15.6° for the Nurotron lateral wall EA (Table 1). Among the 12 ears, 11 EAs realized full tympanic scalar positioning, only one ear (8%) with perimodiolar EA presented scalar deviation at 180–210° (Figure 2B).

The maximum cross-section and mastoidectomy sizes were not significantly different between the manual and the robotic-assisted insertion side in the five infants (*Z* = −0.80904, *p* = 0.438; *Z* = −1.07872, *p* = 0.313, respectively) or in all cases (*Z* = 0, *p* = 1; *Z* = −1.25794, *p* = 0.219, respectively) (Table 2). Furthermore, the average mastoidectomy size in the infants (1,925.6 ± 435.2 mm^3^, *n* = 10) was about one-third of the size in the adult (5,584.0 ± 33.9 mm^3^, *n* = 2). The surgical cross-sections in the infants (166.9 ± 18.7 mm^2^, *n* = 10) were about half of the size in the adult (333.6 ± 65.1 mm^2^, *n* = 2). The anterior-posterior and superior-inferior distances of the posterior tympanotomy, and the facial recess distance from the skin in the infants were about 70% of those in the adult.

**Table 2:** Anatomic parameters of cochlea and mastoidectomy in infant and adult recipients.

**Age at surgery**	**Cochlea size (mm)**			**Surgical technique**	**Mastoidectomy size**				
	**Length**	**Width**	**Height**		**Maximum cross-sectional area (mm^**2**^)**	**Anterior–posterior distance (mm)**	**Superior–inferior distance (mm)**	**Facial recess distance from skin (mm)**	**Mastoidectomy volume (mm^**3**^)**
13 mo	**9.3**	**6.5**	**3**	**R**	**163.3**	**18.1**	**11.3**	**23.0**	**1,542.0**
	8.9	5.9	3.1	M	171.2	19.2	11.3	22.7	1,938.0
13 mo	**8.7**	**6.5**	**3.3**	**R**	**118.7**	**14.8**	**10.3**	**25.8**	**1,325.0**
	8.8	6.7	3.3	M	169.5	17.3	12.4	24.2	1,892.0
10 mo	**9.5**	**6.9**	**4.0**	**R**	**164.4**	**18.8**	**9.9**	**25.3**	**1,612.0**
	9.1	6.8	3.8	M	190.9	20.1	11.6	23.0	1,929.0
42 mo	**9.1**	**6.6**	**3.3**	**R**	**179.0**	**17.2**	**11.7**	**26.5**	**2,587.0**
	10	6.8	3.2	M	169.2	20.2	10.1	25.7	2,699.0
12 mo	**10.4**	**7.3**	**2.9**	**R**	**172.6**	**17.6**	**13.0**	**23.6**	**2,031.0**
	9.9	6.7	3.3	M	169.7	18.6	11.5	22.2	1,701.0
Average in children	9.4 ± 0.6	6.7 ± 0.4	3.3 ± 0.3		166.9 ± 18.7	18.2 ± 1.6	11.3 ± 1.0	24.2 ± 1.5	1,925.6 ± 435.2
*39 yr*	***9.5***	***6.9***	***3.0***	***R***	***379.6***	***29.4***	***16.7***	***32.1***	***5,560.0***
	*9.1*	*7*	*2.8*	*M*	*287.5*	*25.0*	*14.1*	*31.7*	*5,608.0*

*Age column: mo, month; yr, year. R, inserted with robot assistance. M, inserted manually. Boldface indicates the robot-assisted side. Italics indicates the adult case*.

**Figure 4:**
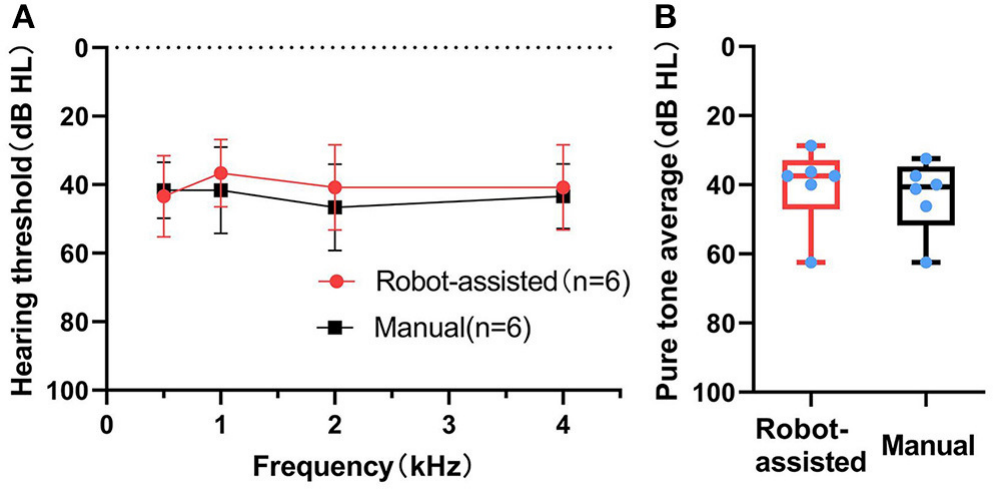
(Behavior) Pure-tone audiometry with CI after 6–9 months. Pure tone audiometry (PTA) thresholds **(A)** and pure tone average **(B)** in the robot-assisted (*n* = 6) and manual insertion (*n* = 6) groups 6–9 months after first mapping.

All cases benefited from CI. The average aided pure-tone audiometry (PTA) with CI was 42 ± 10.6 dB HL about 6–9 months after implantation. The average aided PTA was not different between the manual and the robotic-assisted insertion side (40 ± 11.5 dB HL vs. 43 ± 10.4 dB HL, *n* = 6, *Z* = −1.36083, *p* = 0.250) (Figure 4). In the adult recipient, the monosyllabic word recognition score, disyllabic word recognition score, and sentence recognition score (SRS) were 48, 38, and 82% in the robotic-assisted insertion side, and 38, 36, and 88% in the manually inserted side, respectively.

## Discussion

Currently, the robotic assistance techniques in CI mainly focus on two surgical manipulations: the minimally invasive approach to the inner ear (called direct cochlea access) (12, 27, 28), and minimally invasive EA insertion (14, 15, 29, 30). Different application scenarios with surgical robots have led to various designs for the robotic systems. To gain middle ear access, HEARO® and micro-stereotactic systems use task-specific robotic techniques to perform high-precision automatic drilling procedures (12, 13). Subsequent EA insertion is carried out manually through the narrow tunnel, which might increase the occurrence of tip fold-over (31). Because the minimal invasive EA insertion and accurate scalar positioning attached more relevance to good audiological outcomes, RobOtol® is designed to replace manual insertion with robotic insertion while all other steps are the same as routine surgical procedures. This small change makes the learning curve shorter, and training for this device only takes a few hours for an experienced otologist.

From childhood to adulthood, the mastoid grows in terms of length, width, and depth, and growth and development reach an initial plateau by the age of 7 years for all dimensions (32). Smaller mastoidectomy size mainly limits the translation and inclination of the surgical tool and introduces challenges for robot-assisted manipulations. Therefore, all current reports of robotic systems for cochlear implantation were studied in adults. Because the children are the leading candidates for CI in most countries, the possible and safe application of robotic systems in pediatric CI needs to be thoroughly investigated.

In this series, as all children were younger than 4 years, their surgical zone (cross-sectional area of mastoidectomy) was about 18 × 11 mm, which was about half of the adult (27 × 15 mm). Under this anatomical limitation, RobOtol® was successfully applied in all children with one try of EA insertion, as no additional local trauma or complications occurred. The postoperative radiological image revealed no difference in the mastoidectomy size between the two sides, which means that routine mastoidectomy is sufficient to allow robotically realize EA insertion. These anatomical data could also inspire the development of related robotic tools.

While preparing for insertion of the EA, the Med-EL array required more time to reach the optimal axis. Its ultra-soft features and the clamping site, which is further from the tip, are considered to be the main causes. While inserting the EA, the duration for perimodiolar EA insertion was shorter because the AOS technique for this type of EA needs cooperation as the stylet is manually held and retracted, which could not well control speed as fully robotic manipulation. We had to apply manual-robotic cooperation mode for AOS technique because the fully automatic AOS procedure requires more degrees of freedom, which inevitably enlarges the instrument and requires further validation.

The insertion depth was no different on the two insertion technique sides with the same EA. Full tympanic scalar insertion was realized for all lateral wall EAs, under either manual or robotic technique. For the perimodiolar array, full tympanic scalar insertion was all realized with the robotic technique, but one scalar deviation was observed at 180–210° with the manual technique which might be caused by excessive force from the discordance of the two-handed AOS technique. It seems that robotic assistance could overcome this discordance with good mastery of this technology. However, some actions are not totally automatic, and enough and good training was indispensable.

Evaluation of audiological outcomes is more difficult in infants because the speech discrimination score, considered to be the main auditory rehabilitation index, could not be evaluated. Alternatively, the aided behavioral audiometry was studied for these infants. The average threshold at 0.5, 1, 2, and 4 kHz at 6–9 months after first mapping was not different between the two sides. In the ear with scalar deviation, the threshold did not show an evident difference from the contralateral ear. The hearing preservation by robot-assisted EA insertions may be more significant. But the preoperative hearing of the patients in this series was poor (generally > 95 dB HL), which could not be applied for the study of residual hearing.

The introduction of the robotic system to CI changes some procedures. The micro-stereotactic system extended the average surgical duration (from incision to closure) to 3 h (31), HEARO® extended it to 4:05 h (33), while RobOtol® only increased duration by about 10–15 min. If for safety considerations, the minimally invasive approach is aborted, converting to a traditional approach might need more time. For RobOtol®, as the routine surgical workflow was barely disturbed, the additional surgical time required could be explained by the following three points. First, RobOtol® changes the regular surgical layout, as the lateral or front side of the surgeon is completely occupied, leading to interference with the microscope and nurse, and requiring repositioning, so that introducing an exoscope could solve part of the problem (34). Second, the high precision of the robotic arm limits its operating space to a 20 cm range thus the comfortable positioning of the robotic arm in the vicinity of the mastoidectomy is extended by ~3.5 min. Lastly, the positioning of the EA at the entry point to the cochlea takes ~4 min, and the subsequent insertion process is a slow and steady process lasting ~3.3 min on average in our study.

Though following up for a longer period than previously reported studies (16, 17), the small case number and lacking more audiological outcomes in children are still the main limitations of the present investigation; however, the current preliminary results, that robotic-assisted insertion seems to lead to less scalar deviation, encouraged us to carry out a prospective, double-blind, randomized trial for robotic EA insertion (ChiCTR2000036534). Additionally, the realization of a fully robot-assisted AOS technique for the perimodiolar EA needs further development to reduce the influence of the biases from manual manipulation. The high-level evidence of the audiological benefits of this advanced technology will be presented soon. Anyway, this preliminary study might mean the arrival of the era of robot-assisted surgery in all ages of CI recipients.

## Conclusion

The RobOtol® system can safely realize robot-assisted EA insertion for pediatric recipients and can deliver all types of the electrode array. Moreover, the robot-assisted insertion might lead to less intracochlear damage thus potentially improving the long-term audiological outcome, though more evidence needs to be gathered to clarify this. This study serves as a foundation for more research on robotic technology in pediatric cochlear implantation and marks the beginning of a new era in cochlear implantation.

## Data Availability Statement

The original contributions presented in the study are included in the article/supplementary material, further inquiries can be directed to the corresponding author/s.

## Ethics Statement

The studies involving human participants were reviewed and approved by the ethics committee of Shanghai Ninth People's Hospital affiliating to Shanghai Jiaotong University School of Medicine (SH9H-2020-T166-1). Written informed consent to participate in this study was provided by the participants' legal guardian/next of kin.

## Author Contributions

JP, HJ, and YC designed the study. YL, OS, and HW validated the design. JP, HJ, HT, YC, ZZ, MJ, YL, and HW conducted experiments. JP, HT, and YC acquired data and visualized the results. JP and WG drafted the manuscript. OS, HJ, and HW analyzed data and reviewed the manuscript. All authors have read and approved the final version of the manuscript.

## Conflict of Interest

The authors declare that the research was conducted in the absence of any commercial or financial relationships that could be construed as a potential conflict of interest.
